# Half-Yearly Report of Cases in Midwifery, Which Have Occurred in the Northern District of the London and Southwark Midwifery Institution

**Published:** 1828-07

**Authors:** C. Waller

**Affiliations:** One of the Consulting Accoucheurs to the above Institution, and Lecturer on Midwifery at the Medical School, 58, Aldersgate-Street.


### MIDWIFERY.

*Half-yearly Report of Cases in Midwifery, which have occurred in the Northern District of the London and Southwark Midwifery Institution.* By C. Waller, Esq. one of the Consulting Accoucheurs to the above Institution, and Lecturer on Midwifery at the Medical School, 58, Aldersgate-street.

It having been intimated to me that a report of the cases occurring in the northern district of our institution would be acceptable for publication in your Journal, I lose no time in complying with the request: at the same time, however, observing, that it cannot be considered a perfect report, from my not being aware that it would be published, and consequently from my neglecting those means which would have insured a more correct return. If, with these imperfections, it is worthy your notice, it is perfectly at your service.

| Number of Women delivered. | Sex of Children. Males. | Females. | Born alive. | Still-born. | Presentation. |
|---|---|---|---|---|---|
| December, 1827. | | | | | |
| 27 | 15 | 12 | 25 | 2 | 26 Natural<br>1 Breech |
| January, 1828. | | | | | |
| 34 | 18 | 16 | 34 | 0 | 32 Natural<br>2 Breech |
| February. | | | | | |
| 33 | 18 | 15 | 32 | 1 | Natural |
| March. | | | | | |
| 27 | 18 | 9 | 27 | 0 | Natural |
| April. | | | | | |
| 22 | 9 | 14 (1 twin case) | 22 | 1 | Natural |
| May. | | | | | |
| 22 | 14 (1 twin case) | 9 | 23 | 0 | Natural |
| Total, 165 | 92 | 75 | 163 | 4 | |

*Remarks.*—Of the four still-born children, two were born between the sixth and eighth month of utero-gestation, and the other two were first children, in which the breech presented : in one there was plenty of room, but a great deficiency of uterine action; in the other the pelvis was considerably contracted, so that great difficulty was experienced in extricating the head. In the first case I exhibited two doses of the secale cornutum, the first of which was ejected from the stomach, and the latter did not appear to have any effect. It may, however, be observed, that the medicine had been exposed to a damp atmosphere, and had been dried before a fire, which probably was injurious to it.

In one case, hemorrhage to a serious extent occurred, but it was effectually checked by the liberal use of cold water and the employment of external friction: and here I cannot help expressing my conviction of the efficacy of *external friction,* when promptly and properly applied, believing that it would, in almost every instance, supersede the necessity for the introduction of the hand *within*; an operation always productive of pain, and not altogether unattended with risk. The patient was twenty-seven years of age, and had been confined four times previously, and had usually been subject to large losses of blood. Her getting-up was protracted in consequence of her suffering severely from that irritable state of the head and of the bowels, so frequently the result of profuse hemorrhage. The combination of calomel with large doses of opium appeared to be very serviceable.

In one female, fever of the low type occurred, after a very natural and easy labour. This patient was miserably poor, and had been shamefully neglected by a creature calling herself a nurse. There was excessively increased action, with diminished power, the pulse rising to 160, and continuing above 120 for several days; the head much disturbed; the countenance alternately flushed and pallid; the bowels at first constipated, a horribly offensive diarrhœa afterwards came on, the fæces passing involuntarily, as well as the urine; the uterine discharge was very offensive, and there was no secretion of milk. She was also at times exceedingly distressed with vomiting. There was at no time any abdominal pain, which circumstance alone led me to hope for a favorable result. The tongue throughout indicated great intestinal irritation, being either morbidly red or brown, usually moist, being at no time covered with that hard dry crust which we sometimes observe in fevers of this description. It should also be noticed, that she had, throughout her complaint, that peculiar sharp manner of talking so disagreeable to the accoucheur, inasmuch as it usually portends the most imminent danger. The treatment of this patient was very varied, being adapted to the symptoms as they arose. Her bowels being at first confined, the indication was obviously to relieve them, which was done with some difficulty. The stomach irritation was relieved by small doses of the Soda Tart. and the liberal use of the effervescing draught; which latter was exceedingly refreshing to her. Calomel and opium, by producing sleep, assisted in the cure, and, as soon as we could safely do it, the judicious introduction of food into the stomach. On the supervention of the diarrhœa, the pulse fell from 160 to 130, and consequently I did not attempt to restrain it suddenly, especially as the symptoms of exhaustion were not increased by it. When it was thought right to check it, (for it occurred more than once during her illness, small doses of Conf. Opii, with Conf. Aromat. dissolved in peppermint water, answered the purpose very well. Never did I have an opportunity of observing the sudden effects of cleanliness as in this case. On my visit one day, I found her apparently in an almost desperate condition, when I was requested to examine a sore on her nates, which I was told was extending. On inspection, I saw a very irritable sloughy sore, which, if not altogether produced by, was certainly aggravated in consequence of, the filthy state she was allowed to remain in. I directed thorough ablution, and the very next day, to my utter astonishment, she was sitting at the fire dressed. From this time there was no relapse.

A case of fatal apoplexy occurred in a young woman, only twenty-three years of age, who miscarried, for the seventh time, at about the seventh month of gestation. The labour was a very easy one, and she appeared, for the first two or three days, to be doing remarkably well: at the end of this time she was seized with great pain in the head, and a loss of motion in one half of her body; the mouth also was much drawn on one side. The pain, and the distortion of the face, were completely relieved by blood-letting and the application of leeches, followed by a blister; she was then salivated. The muscles of deglutition, however, became paralysed, so that no nourishment could be administered; nor had she the power of bringing up a very tough viscid mucus, with which the mouth and fauces were covered. I attempted to excite vomiting by tickling the fauces, but the sensibility of these parts seemed to be lost. She sank, as far as my recollection serves me, (for I omitted to take a memorandum,) on the seventh or eighth day after delivery. No examination of the body was allowed. The friends of this female informed me that she experienced two slight attacks during her pregnancy.

In one case the use of the forceps was required. The patient, a stout young woman, ætat. twenty-eight, was taken in labour with her first child, on the 2d of March: the pains continued pretty regular, though not severe, till the 5th, when I was requested to see her. The os uteri was at this period only half dilated, and the parts of generation rigid; the liquor amnii had been discharged about twenty-four hours; the pains feeble; the head situated completely above the brim of the pelvis. She did not appear to be suffering at all from fever, but, being of a strong habit, I ordered her to lose twelve ounces of blood, to have an injection administered, and afterwards a full dose of opium. At two o'clock in the morning of the 6th, I was again desired to visit her, and found the os uteri very nearly dilated, the parts of generation much more moist, the pains strong, and the head descending; every thing, in fact, promising a speedy termination to the labour. At ten o'clock I again visited her, and, finding little or no advance had been made, I determined to deliver with the forceps. Considerable difficulty was experienced in the extraction of the head, which was of a very large size. The infant, however, lived, and the mother had a good getting up.

An inflammatory adhesion of the placenta occurred in one instance, attended with considerable loss of blood: on removing the placenta, the discharge immediately ceased.

It was my intention to have appended to this report a few select cases which have lately fallen under my observation, from other sources than that of the institution: time, however, will not at present allow of my doing so, and I must therefore defer it to some future opportunity.

*Bartholomew Close; June 5th,* 1828.

---

